# Video-Assisted Thoracoscopic Surgery Repair of Diaphragmatic Hernia With Prolene Mesh After Microwave Ablation

**DOI:** 10.7759/cureus.80866

**Published:** 2025-03-20

**Authors:** Audrey Poh Poh Tan, Cynthia Ming Li Chia

**Affiliations:** 1 Cardiothoracic Surgery, Singapore General Hospital, Singapore, SGP

**Keywords:** complication, diaphragmatic hernia, hepatocellular carcinoma, microwave ablation, repair, video-assisted thoracic surgery

## Abstract

Microwave ablation (MWA) is one of the minimally invasive techniques utilized in the treatment of hepatocellular carcinoma (HCC). MWA has become an increasingly popular and comparable alternative therapy to radiofrequency ablation (RFA) with similar major complication and mortality rates. Diaphragmatic hernia is a rare complication of MWA. Current literature has very few reports on the management of such complications. This article describes a case of multifocal HCC treated with MWA, complicated by pleural effusion and diaphragmatic herniation of the omentum into the right intrathoracic cavity. The surgical repair of the diaphragmatic defect was performed via video-assisted thoracoscopic surgery (VATS) approach with Prolene mesh reinforcement. Further studies are needed for evaluation of the best surgical approach to manage post-MWA-related diaphragmatic hernias.

## Introduction

Microwave ablation (MWA) is one of the minimally invasive techniques utilized in the treatment of hepatocellular carcinoma (HCC). This thermal ablation technique destroys tumor cells by coagulative necrosis while causing minimal damage to the surrounding tissues [1]. A 2.6%-4.6% major complication rate is associated with MWA [2], largely subdivided into vascular, biliary, mechanical, and infectious complications [3]. This includes pseudoaneurysm, intraperitoneal hemorrhage, biliary stenosis, bowel perforation, and liver abscess [1-3]. Diaphragmatic hernia is a rare complication of MWA, seen in only up to 0.049% of cases [4]. Current literature has very few reports on the management of such complications. We present a case of multifocal HCC requiring multiple treatments, including multiple MWAs, which was eventually complicated by a diaphragmatic hernia, and discuss the management of this complication.

## Case presentation

**Figure 1:**
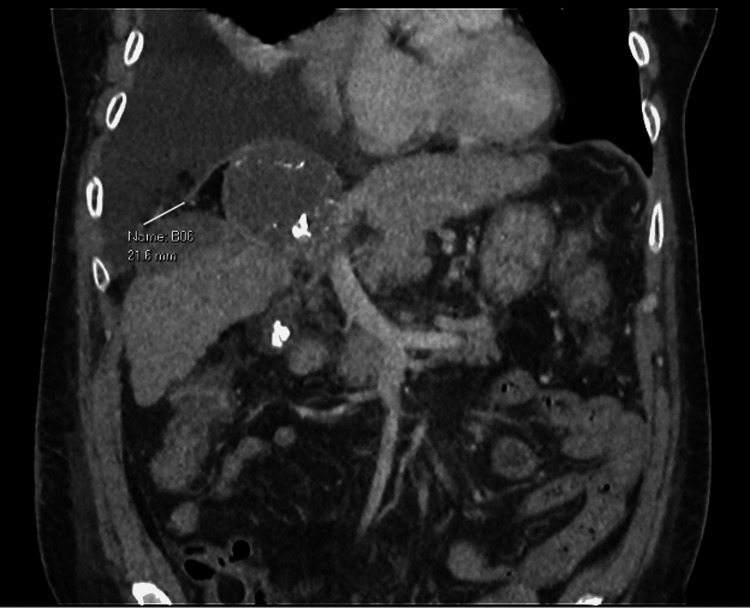
Surveillance CT liver done in October 2024 demonstrating a 2.2 cm right diaphragmatic defect (adjacent to the treated segment VIII/V lesion), with herniation of fat into the right lower hemithorax, with concerns of a possible pleuro-peritoneal fistula.

A 63-year-old female with a 25 pack-year smoking history and asthma first presented with right hypochondrium pain and fever in 2020. She was evaluated and diagnosed with Child’s A liver cirrhosis secondary to hepatitis C complicated by multifocal HCC in segments IV and VIII. She underwent multiple interventions, including Y90 injection into the right hepatic artery, selective embolization of the segment IVa artery, four transarterial chemoembolization procedures in 2020, and stereotactic body radiation therapy to the liver in 2021. On subsequent surveillance scans in January 2023, there was a notable progressive enlargement of the segment VIII/V hepatoma. The patient hence underwent three MWAs from March 2023 to August 2024. This was unfortunately complicated by a 7.4 x 6.6 cm segment II liver abscess for which percutaneous drainage with an 8-French Navarre catheter over a trocar needle was performed in September 2024. On repeat surveillance imaging with computed tomography (CT) liver three weeks later, a 2.2 cm right diaphragmatic defect (adjacent to the treated segment VIII/V lesion) with herniation of fat into the right lower hemithorax and a large right pleural effusion was noted, with concerns of a possible pleuro-peritoneal fistula (Figure 1).

The patient underwent image-guided right chest drain insertion with an 8-French drainage catheter, which was subsequently upsized to a 12-French Skater drainage catheter due to a blocked drain. Investigations for the pleural fluid were unyielding and transudative in nature (Table 1). Bacterial infection was excluded with a negative gram stain and culture and 90% lymphocytes, and pleural tuberculosis was excluded with a negative acid-fast bacilli smear and a normal adenosine deaminase level at 4.4 U/L. Cytology demonstrated mixed inflammatory cells with no malignant cells present. Drain output remained mainly serous throughout. Percutaneous drainage of segment II liver collection also yielded negative fluid gram stain and cultures. 

**Table 1:** Pleural fluid analysis demonstrating no bacterial or tuberculosis infection and no evidence of malignant cells.

Pleural fluid analysis		Normal reference range
Appearance	Yellow and clear	
Protein total	18.0 g/L	
Specific gravity	1.014	
Glucose	8.6 mmol/L	
Lactate dehydrogenase	72 U/L	
Neutrophil %	10	<1
Lymphocyte %	90	20-23
Cell count	240 cells/mm^3^	<1000 cells/mm^3^
Gram stain smear	No organism seen	
Gram stain culture	No bacterial growth	
Acid-fast bacilli smear	No acid-fast bacilli seen	
Adenosine deaminase	4.4 U/L	0.0-30.0 U/L
Cytology	Mixed inflammatory yield. No malignant cells were seen.	

**Figure 2:**
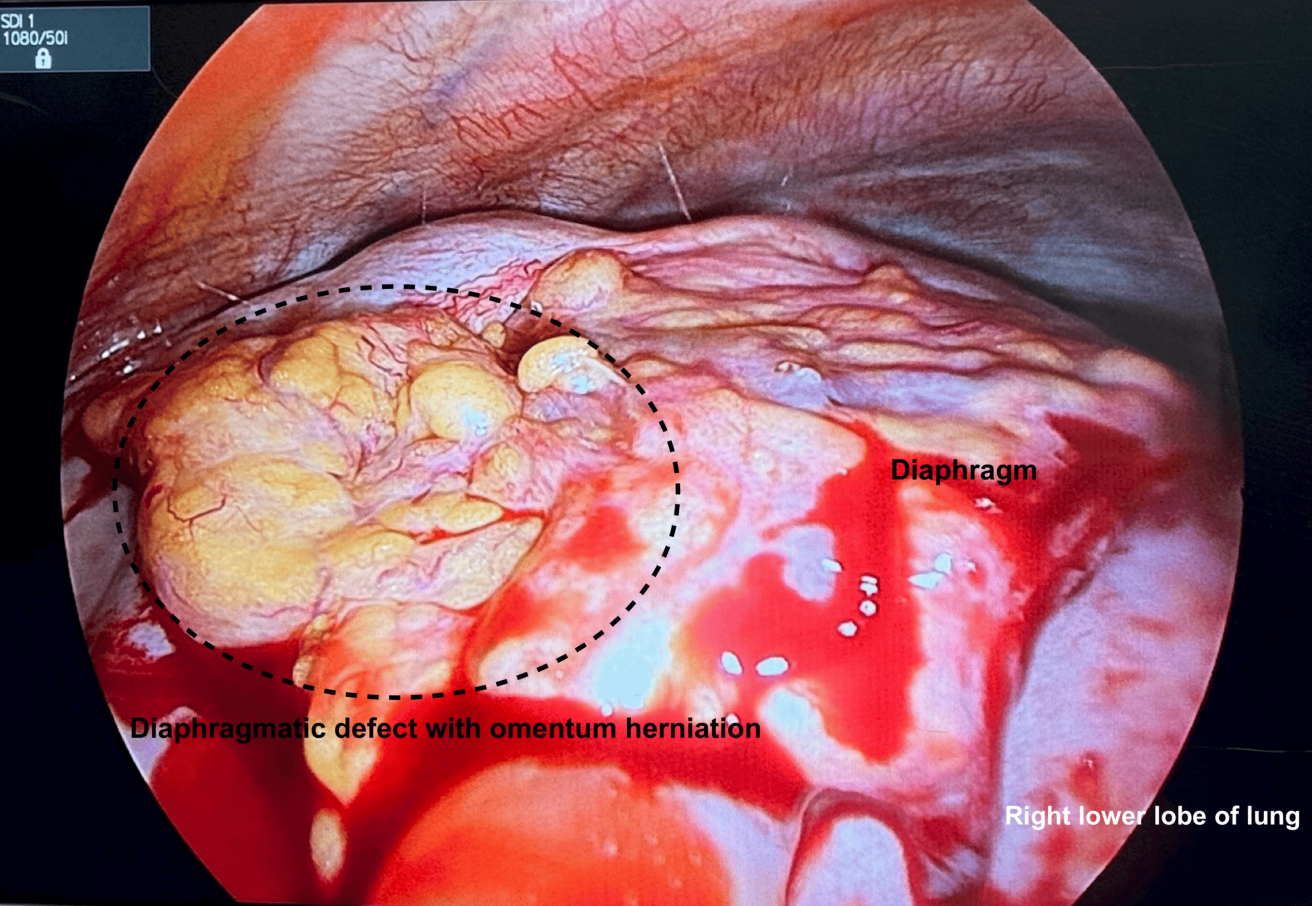
Intraoperative imaging of the 4.5 x 4.0 cm diaphragmatic defect with the omentum herniating through and plastered to the diaphragm.

The patient was referred to the cardiothoracic department for definitive management of the diaphragmatic hernia. She underwent right video-assisted thoracoscopic surgery (VATS), excision of herniated omentum, repair of diaphragmatic defect, and application of Prolene mesh to the diaphragm in October 2024. Intraoperatively, a 4.5 x 4.0 cm diaphragmatic defect was noted with the omentum herniating through and plastered to the diaphragm (Figure 2). 

**Figure 3:**
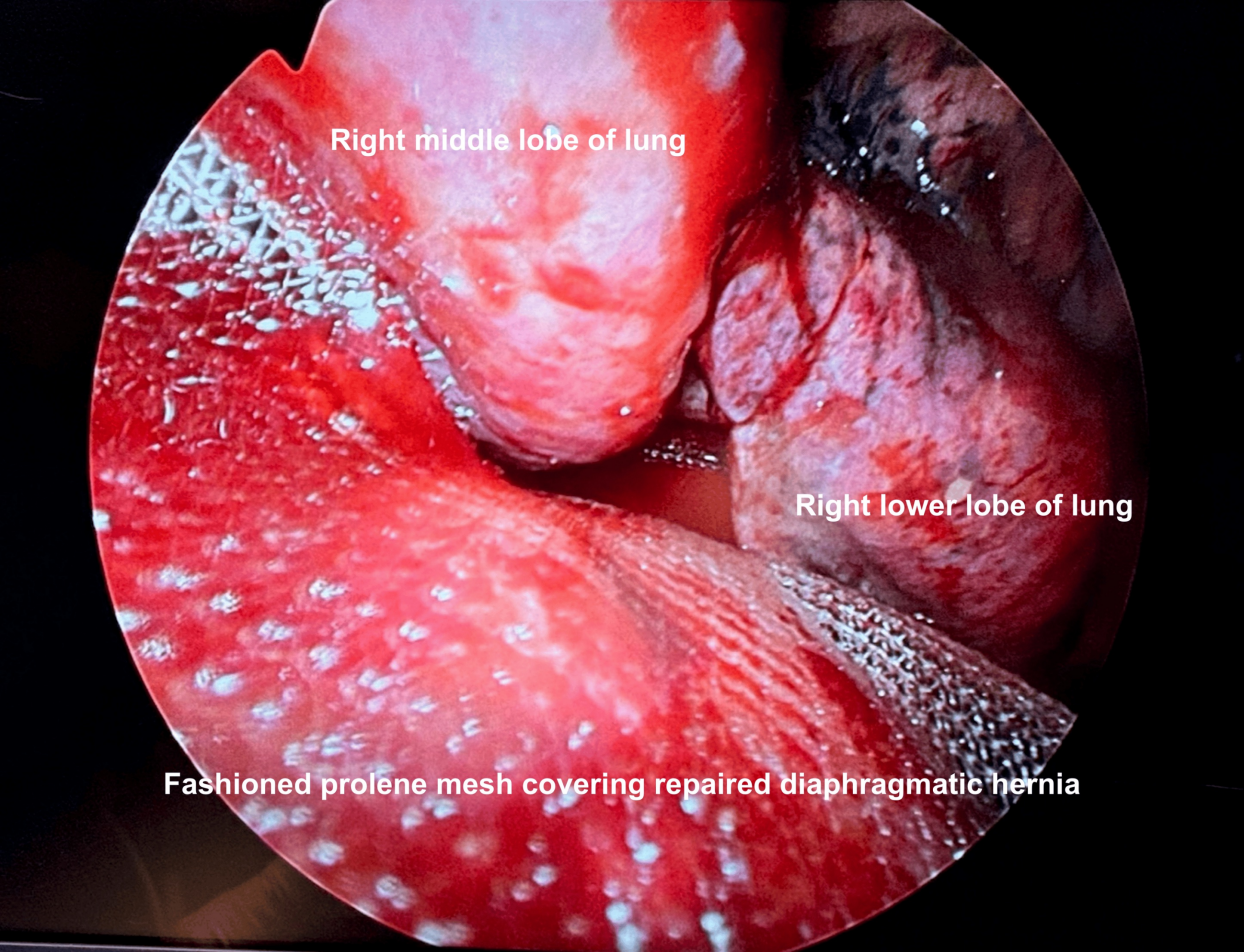
Intraoperative imaging demonstrating fashioned Prolene mesh to fit the diaphragm after diaphragmatic hernia was repaired and the right lower lobe of the lung was freed from the diaphragm.

The right lower lobe of the lung was also adherent to the diaphragm posterolaterally. The omentum was excised, and primary repair of the diaphragm was performed using Stratafix PDS 3/0. Prolene mesh with gentamicin was then fashioned to fit the diaphragm for reinforcement as seen in Figure 3, and ProTack™ (Medtronic, Minneapolis, MN) was used to tack the Prolene mesh to the chest wall. The air leak test showed minimal leak.

**Figure 4:**
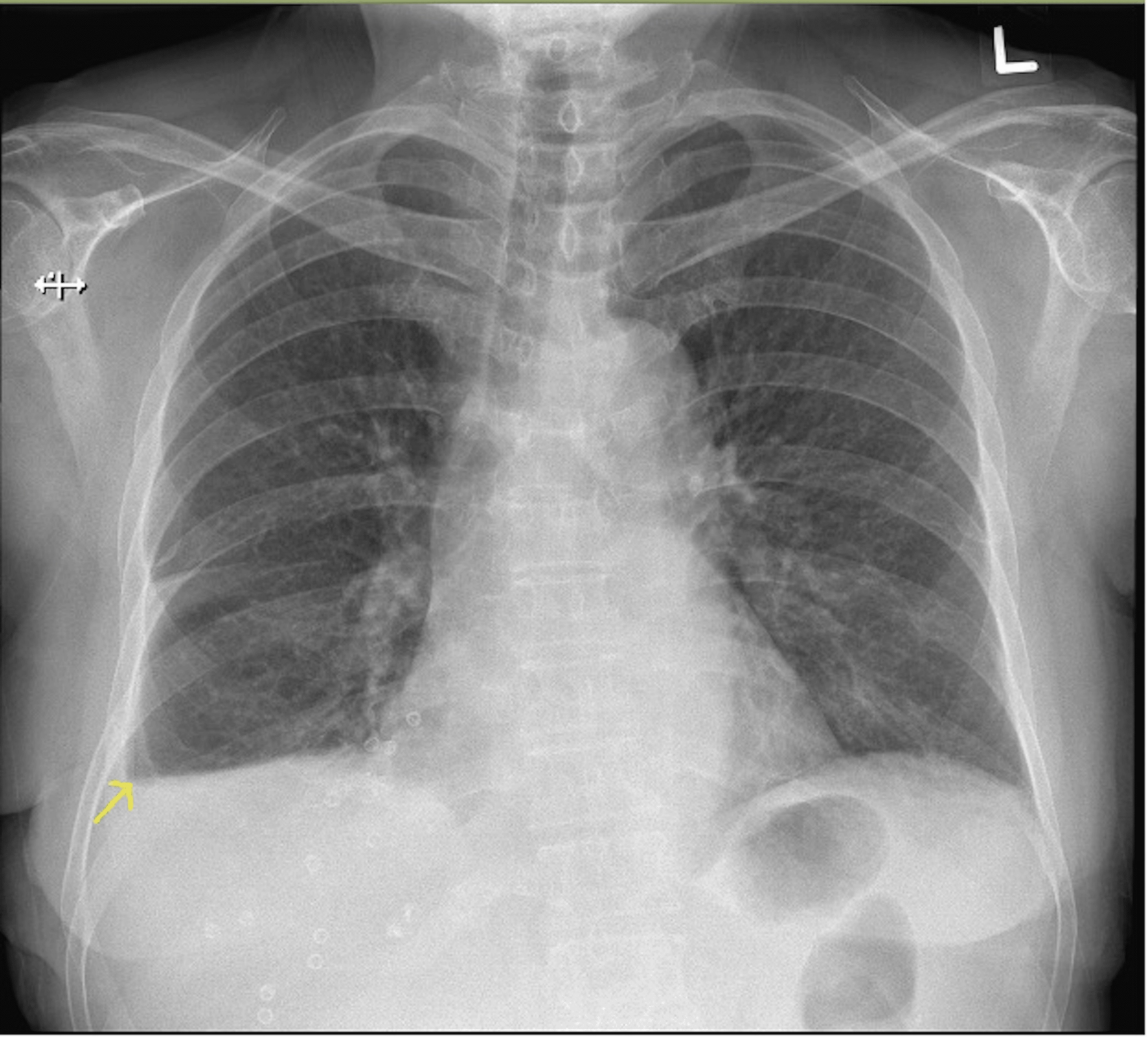
Chest X-ray one month postoperatively demonstrating minimal right pleural effusion.

The patient had an unremarkable postoperative recovery. She was started on bilevel positive airway pressure ventilation with minimal settings of inspiratory positive airway pressure at 6 cm H_2_O and expiratory positive airway pressure at 5 cm H_2_O with 21% fraction of inspired oxygen for five days. She was escalated to diet by postoperative day (POD) 2. Chest drain was kept on active suction at -20 cm H_2_O, converted to passive drainage on POD 3, and removed on POD 4. Chest X-ray one month postoperatively was stable with minimal pleural effusion (Figure 4).

**Figure 5:**
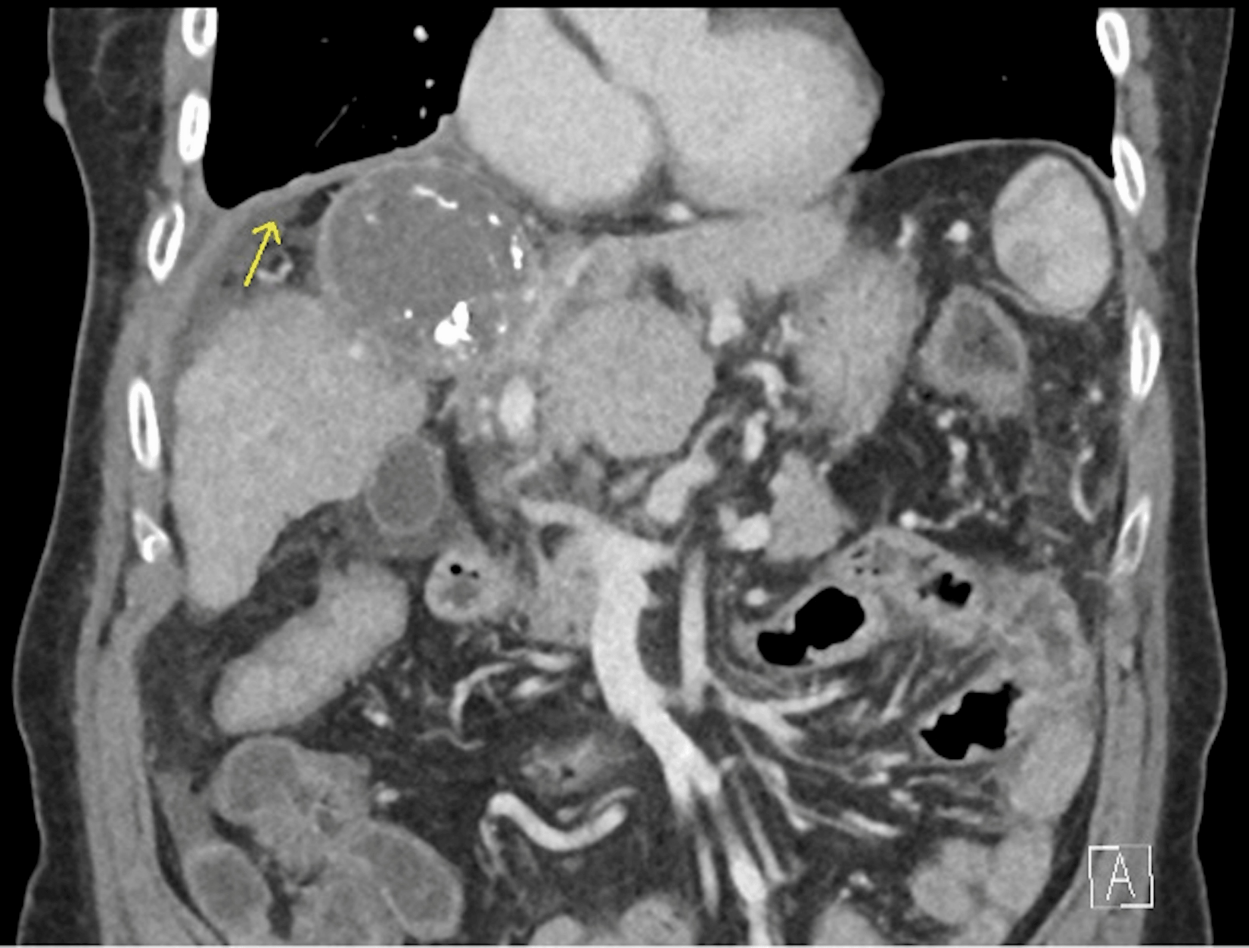
CT liver performed six weeks post-diaphragmatic hernia repair demonstrating intact right diaphragmatic repair with no recurrence of herniation.

CT liver repeated six weeks postoperatively showed an intact right diaphragmatic hernia repair with no recurrence of herniation and only trace right pleural effusion (Figure 5).

## Discussion

Local ablation is commonly considered the first-line treatment in early-stage HCC patients who are not suitable surgical candidates. MWA has become an increasingly popular and comparable alternative therapy to radiofrequency ablation (RFA). Studies have shown that the major complication and mortality rates between MWA and RFA are largely comparable, with no statistically significant difference [2,5,6].

Diaphragmatic injury from local ablation therapies can range from a small diaphragmatic defect to bowel herniation and potentially life-threatening complications. Liver tumors located close to the diaphragm are expected to be at a higher risk of a resultant treatment-related diaphragmatic injury. This is consistent with the common factor seen among the reported cases of diaphragmatic herniation, wherein the treated lesions were in segments VII and VIII. The main lesion requiring repeated ablations in our described case was also in segment VIII.

Thermal damage to the diaphragm may lead to an inflammatory response and resultant fibrosis that eventually weakens the diaphragmatic muscle fibers and causes a late-onset diaphragmatic defect. The associated poor liver function impairs adequate wound healing, and the accompanying complications, such as ascites and pleural effusion, further contribute to tissue damage [7]. 

Macmillan et al. described a case of MWA for HCC complicated by a diaphragmatic herniation of the ascending and transverse colon and the resultant perforation of intrahernial transverse colon requiring emergency laparotomy, extended right hemicolectomy, ileostomy creation, and closure of the diaphragmatic defect [8]. Nagasu et al. described six cases of diaphragmatic perforation after RFA, and all cases were treated by surgical laparotomy and a simple suture of the diaphragmatic defect [9]. The only other described minimally invasive surgical management of MWA-related complication of diaphragmatic defect was performed via intra-abdominal laparoscopic approach and utilized a Dacron heart patch for repair of the 1 cm diaphragmatic defect [10]. Our case is the first case in the literature to describe a VATS approach for repair of a large diaphragmatic hernia and reinforcement with Prolene mesh after primary suture repair of the diaphragmatic defect. The added benefit of our described minimally invasive approach is that Prolene mesh is likely more cost-effective than a Dacron heart patch or a Gore-Tex patch.

There have been various techniques described in the literature to reduce the risks of thermal injury and MWA-related complications, especially in cases of HCCs located close to the gastrointestinal tract or the diaphragm. This includes the creation of artificial ascites or pleural effusion to act as a thermal blanket and ensuring adequate separation of the ablation zone from the adjacent organ [11]. Other factors of essence include preprocedural evaluation of patient factors such as risk factors for infection, coagulation profile, tumor size, number, and location, and consideration of separate ablation sessions for large or multiple ablations [7]. 

## Conclusions

Diaphragmatic herniation post-MWA is a rare but serious complication. Current literature has demonstrated a myriad of approaches in the management and surgical repair of such diaphragmatic hernias, but there is currently no consensus on the most optimal method. We describe the VATS approach for repair of a large diaphragmatic hernia and reinforcement with Prolene mesh after primary suture repair of the diaphragmatic defect. Further studies are needed for evaluation of the best surgical approach to manage post-MWA-related diaphragmatic hernias.
